# Symmetric left-handed split ring resonator metamaterial design for terahertz frequency applications

**DOI:** 10.1038/s41598-023-49202-1

**Published:** 2023-12-09

**Authors:** Tayaallen Ramachandran, Mohammad Rashed Iqbal Faruque, K. S. Al-mugren

**Affiliations:** 1 Space Science Center (ANGKASA), Universiti Kebangsaan Malaysia, UKM, 43600 Bangi, Selangor Malaysia; 2 Physics Department, Science College, Princess Nourah Bint Abdulrahman University, Riyadh, Saudi Arabia

**Keywords:** Electrical and electronic engineering, Characterization and analytical techniques

## Abstract

This work focused on the novel symmetrical left-handed split ring resonator metamaterial for terahertz frequency applications. A compact substrate material known as Silicon with a dimension of 5 µm was adopted in this research investigation. Moreover, several parameter studies were investigated, such as clockwise rotation, array and layer structure designs, larger-scale metamaterials, novel design structure comparisons and electric field distribution analysis. Meanwhile, two types of square-shaped metamaterial designs were proposed in this work. The proposed designs exhibit double and single resonance frequencies respectively, likely at 3.32 and 9.24 THz with magnitude values of − 16.43 and − 17.33 for the first design, while the second design exhibits a response at 3.03 THz with a magnitude value of − 19.90. Moreover, the verification of these results by adopting High-frequency Structure Simulator software indicates only slight discrepancies which are less than 5%. Furthermore, the initial response of the proposed designs was successfully altered by simply rotating the design clockwise or even increasing the dimension of the design. For instance, the first resonance frequency is shifted to the lower band when the first proposed design was rotated 90°. On the other hand, by increasing the size of the metamaterial, more than nine resonance frequencies were gained in each symmetric design. Furthermore, the symmetric metamaterial with a similar width and length of 10 µm dimension was adopted for both design structures to construct an equivalent circuit model by utilising Advanced Design System software. Finally, both unit cell designs were utilised to explore the absorption performances which exhibit four and five peak points. Overall, the altering behaviour by changing physical properties and compact design with acceptable responses become one of the novelties of this research investigation. In a nutshell, the proposed designs can be utilised in terahertz frequency which gives optimistic or advantageous feedback and is relatively suitable for the adopted frequency range.

## Introduction

In recent years, the metamaterial structure has been widely used in many application fields such as metamaterial absorbers, specific absorption rate reduction, satellite frequency, wireless communication, military applications, transmissive deflection, focusing, and orbital angular momentum, etc.^1–9^. The metamaterial term is defined as man-made material that is unable to be found in nature. Moreover, because of its unique properties, metamaterial is still explored by researchers around the world. One of the famous applications of this unconventional material is the miniaturisation concept. This is because the miniaturised electronic devices or systems had a big impact on the military, business, and industry even before they arrived. Miniaturisation enabled communications, data handling and processing, and space exploration. On the other hand, metamaterial is also widely used in the terahertz frequency range. The terahertz (THz) regime in the electromagnetic spectrum holds potential for a wide range of critical applications, including environmental sensing, quality control, medical, military applications, astronomy, and so on.

In 2021, Pitchappa et al. proposed an electromechanically reconfigurable microcantilevers integrated complementary metamaterial to function as a frequency‐agile THz bandpass transmission filter^10^. The tunable THz transmission filters are critical for a variety of vital applications including channel selectors in high‐speed wireless communication systems, hyperspectral imagers, and miniaturized spectrometers. Besides that, a new controlling technique of the plasmatic electron packet based on an electric split-ring resonator was proposed by Ahmed et al.^11^. The proposed metamaterial tunnelled structure in this study operates using the THz frequency spectrum as an efficient digital processing filter. Moreover, the tunnelled structure’s array combination was made up of three distinct unit cells. Recently, Anwer^12^ explored a novel terahertz sensor based on a metamaterial absorber. The proposed structure is made up of a rectangular gold metallic resonator positioned atop a polyimide dielectric medium and a metallic board at the bottom. On the other hand, Ma et al.^13^ characterised the electromagnetic response of a planar array of complementary square metal frames produced on a high resistivity Si substrate using THz time-domain spectroscopy, and between the two layers existed a significant ferroelectric layer of strontium titanate. The frequency-dependent magnetic and electric resonances accord perfectly with theory. The authors show that it has two unique reflection dips after reconfiguring the metamaterial by etching a rectangular stripe, whereas the former metamaterial only had one resonance frequency.

Moreover, Ahamed et al.^14^ introduced a double negative bend-headed I-shaped metamaterial-based terahertz optical power splitter to investigate power splitting via backward wave propagation. The first array pattern is a rectangular array configuration that is analytically characterised and proposed as a power splitter in the transverse electric mode. Another cavity array construction was presented in this work based on the proposed rectangular metamaterial array pattern. In 2020, Tetik^15^ introduced a flexible perfect metamaterial absorber and sensor application at terahertz frequencies. The proposed unit cell metamaterial possesses a flexible structure composed of a gold ground plane, GaAs patch, gold resonator and polyimide substrate. Meanwhile, Xu et al.^16^ designed and explored an optically switchable metamaterial absorber in the terahertz regime. The integrated semiconductor Silicon and a periodic array of metallic split-ring resonators make up the proposed metamaterial absorber. In 2021 a perfect metamaterial absorber was proposed by Zhang et al.^17^ for the terahertz frequency range. Mie-resonance-based metamaterials are created in this study using AI_2_O_3_ microspheres of various dimensions The arrays of spheres are created quickly and precisely using a simple procedure.

A multi-band metamaterial absorber operating at terahertz frequencies was proposed by Cheng et al.^18^ in 2014. This work also includes the design, characterisation, and theoretical computation of a high-performance metamaterial absorber. A dielectric spacer separates two metallic layers in the multi-band metamaterial absorber. Meanwhile, Wang et al.^19^ suggested a multiple-frequency-band terahertz metamaterial absorber with adjustable absorption peaks using the surface structure of the toothed resonator. It can alter the number of absorption peaks without increasing its design complexity, which differs from prior research that had to compromise metamaterial design complexity. Furthermore, Duan et al.^20^ analysed and explored the dependence of the metamaterial absorption maxima on the spacer layer thickness and the reflection coefficient of the metamaterial layer obtained in the absence of the ground plane layer. On the other hand, a laser-excited frequency-switchable and polarization-controlled amplitude-tunable terahertz metamaterial absorber was suggested by Li et al.^21^ in 2022. External laser excitation controls the frequency-switchable function, whereas the incidence polarisation angle controls the amplitude-tunable function. Active control of electromagnetically induced transparency in a terahertz metamaterial array with graphene for continuous resonance frequency tuning was suggested by Kindness et al.^22^. By combining metal-coupled resonator arrays with electrically controllable graphene, active resonance frequency tuning of a terahertz metamaterial is achieved.

Even though there have been numerous research works on metamaterial for terahertz frequencies conducted in recent years, there have been limited studies on the miniaturisation concept have been performed for the adopted application field. To get optimal results, substantial research studies with various unique and novel constraint variables are typically necessary. This is because, most of the previously published works either focused on the larger unit or array cell structures, likely in research works from^11–13^. Moreover, the overall analytical investigation of the proposed work also revealed that larger metamaterial design structure exhibits better resonance frequencies and magnitude responses. However, compact designs typically fail to exhibit desired peak points and magnitude values either in lower or higher THz frequencies. On the other hand, almost all the research works discussed in this literature review adopted only a smaller frequency range and therefore able to meet the objectives. Furthermore, typically the researchers adopted simple metamaterial design structures that only produce limited characteristics and performances. Hence this work focused on the analysis of a larger frequency range scale and unique design structure for THz application. Therefore, by introducing not only one but two types of compact symmetric square-shaped split ring resonator metamaterial design structures, this work explores its behaviours and novel performances between the frequency range from 0 to 10 THz. Moreover, several parametric studies with promising and unique outcomes are analysed and explored such as clockwise rotation, array and layer structure designs, larger scale designs, novel design structure comparison, electric field distribution and absorption properties. During the initial analysis, two types of symmetric split ring resonator designs were selected to explore the performance which has different resonance frequencies. Throughout this parametric study, a novel behaviour of altering the responses was gained by simply performing a few analyses by using existing designs instead of changing or synthesising new designs or materials. This becomes the originality of this work where the S21 properties of a distinct metamaterial design can be successfully altered by arranging the unit cell metamaterial in a novel structure. Furthermore, this work also verified the initial S21 results from CST software by adopting two different analytical software likely, HFSS and ADS software and also presented an equivalent circuit model of the proposed designs. In a nutshell, two different novel split ring resonator metamaterial designs with distinct dimensions were proposed successfully to meet and satisfy the objectives of this research investigation.

## Unit cell design and simulation methods

**Figure 1:**
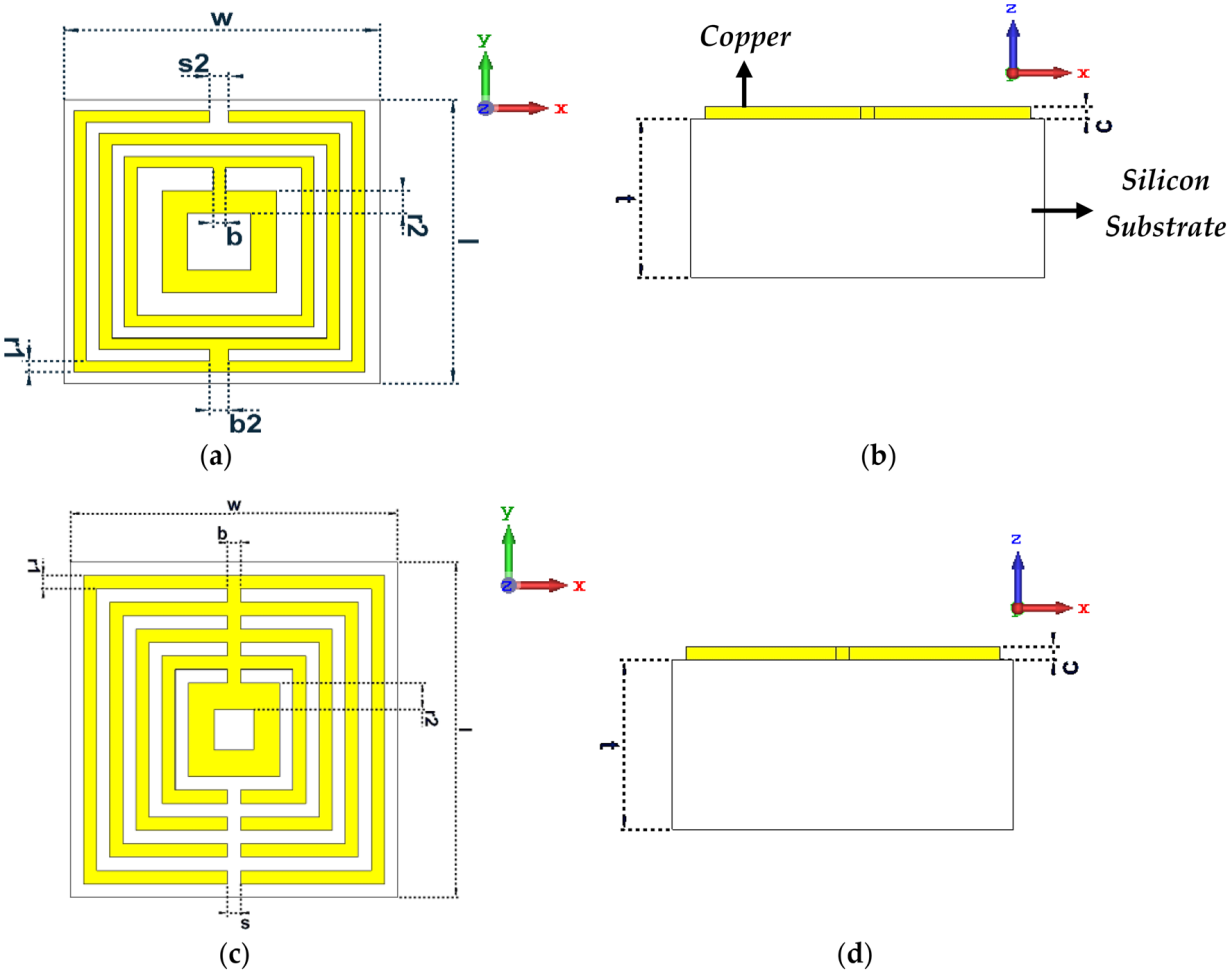
Two types of proposed symmetric square-shaped metamaterial designs adapted from CST software (**a**), (**c**) Top view; (**b**), (**d**) Side view.

This section contains in-depth discussions on how to build the unit cell metamaterial designs. The resonant electromagnetic response of metamaterials, which greatly improves their interaction with terahertz radiation is where their potential for terahertz applications comes from. As a result, metamaterials provide a way to contribute to bridging the so-called terahertz gap. However, the number of resonance frequencies relatively decreases as the overall dimension of the unit cell design becomes smaller. Therefore, this is the main constraint that the researchers faced during the construction of compact metamaterial design for terahertz frequency application. Hence, the selection process of the type of metamaterial design is crucial in this work. Under the influence of an external magnetic field, the induced electromotive force around the square split-ring resonator causes a current to flow from one ring to the other through the inter-ring space. It also has a higher current distribution and a lower resonance frequency. The square split ring resonator features an additional capacitive coupling to the composite structure, allowing for stronger resonance behaviour. In conclusion, two types of square-shaped metamaterial design structures were adapted to analyse the variation in the scattering parameter performances. The first design comprises four split-ring resonator rings on the Silicon substrate material which possesses a dielectric constant (ɛ) of 11.9 and tangent loss (δ) of 0.00025 S/m (as demonstrated in Fig. 1a,b). Silicon substrate materials are commonly employed for cell behaviour studies on microfabricated surfaces as well as microdevices. The utilisation of this type of material has several advantages for the adopted application field. Silicon provides more design and manufacturing freedom than other substrate materials. Furthermore, it is mechanically stable and can be incorporated with electronics on the same substrate. Signal transduction electronics, such as ‘p-‘ or ‘n-‘ type piezoresistive, can be easily integrated with the Silicon substrate, which is excellent for transistors. Furthermore, the melting temperature of Silicon is 1400 degrees Celsius, which is roughly twice that of aluminium. As a result of its high melting point, Silicon is dimensionally stable even at high temperatures. Meanwhile, the second design is composed of five split-ring resonators as shown in Figs. 1c,d. Both designs have a similar dimension of 5 µm × 5 µm and a thickness of 2.5 µm. Meanwhile, 0.2 µm thick split-ring resonators were adopted for both designs except the last ring which adopted 0.4 µm, respectively. Design 1 has ring gaps of 0.2 µm between the first to third rings and a 0.4 µm gap before the fourth ring as illustrated in Fig. 1a. However, Design 4 as shown in Fig. 1c has only 0.2 µm gaps between all the split ring resonators. Meanwhile, both designs possess split gaps and connecting bars with varied dimensions. The split gap acts as a critical constraint in determining the desired properties. This is because the split gap modifications affect inductance and capacitance values. Furthermore, the resonance frequencies are also affected by changes to the split gaps and have a substantial impact on the recommended unit cell design. All the dimension specifications are tabulated in Table 1.

**Table 1:** Dimension specifications of both adopted symmetric metamaterial designs.

Specification	Dimension (µm)
Substrate width, w	5.0
Substrate length, l	5.0
Connecting bar, b	0.2
Connecting bar, b2	0.3
Split gap, s	0.2
Split gap, s2	0.3
First ring, r1	0.2
Last ring, r2	0.4
Substrate thickness, t	2.5
Metamaterial design thickness, c	0.2

**Figure 2:**
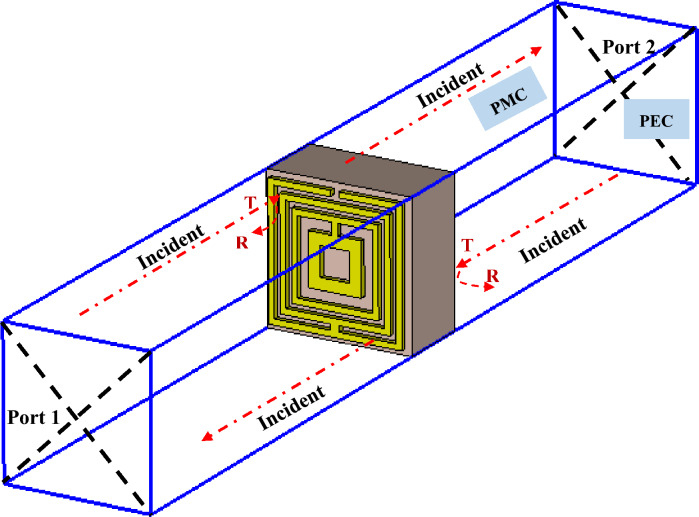
Numerical set-up of Design 1 exported from CST software.

The commonly employed software programme known as Computer Simulation Technology (CST) Microwave Studio 2019 was used to carry out the simulation analyses in this paper. Additionally, a CPU with an Intel (R) Core (TM) i7-10,700 @ 2.90 GHz and 16 GB of RAM were used to execute the simulation. The size of the structure affects the length of time it takes for the simulation to finish; smaller structures take less time than larger-sized symmetric metamaterial designs. The terahertz metamaterials are a type of composite metamaterial that interacts at terahertz frequencies. Hence, in materials research, the terahertz frequency range is often characterised as 0.1–10 THz. Therefore, the same frequency range was adopted in this work to analyse both symmetric designs by utilising various parametric studies. All analytical simulations were performed using a hexahedral mesh and a time-domain solver. In order to assess the findings for transmission coefficient (S21), the unit cell metamaterial design structure was positioned between two waveguide ports as shown in Fig. 2. These ports were placed along the z-axis, indicating a transverse electromagnetic mode. In addition, the y-axis was configured to be a perfect magnetic conductor, while the x-axis was set to be a perfect electric conductor. The structure of the design was altered in accordance with acquiring responses in the chosen frequency range by using the trial and error method. The scattering parameters for the suggested design were generated after the first simulation analysis.

Following this discovery, the effective medium parameters of both metamaterial designs were computed using both S11 and S21 results. As a result, the Robust approach^23–25^ was used to determine various parameters such as permittivity (ε), permeability (μ), refractive index (n), and impedance (z) values using MathWorks MATLAB R2021 software. This software is a sophisticated and powerful numerical computing application with a robust high-level scripting language for scientific and mathematical tasks. The retrieval equations of z, n, ε, and μ are represented by the Eqs. (1)–(5) mentioned below. High-Frequency Structure Simulator (HFSS) software, on the other hand, was used to validate the CST numerical simulation result of the unit cell metamaterial design. Hence, for this analytical simulation, the same proper techniques in CST software were used. corresponding methods in CST numerical simulation were adopted for this software.

1 $$ z \, = \, \pm \surd \left\{ {\left[ {\left( {1 \, + {\text{ S}}11} \right)^{2} {-}{\text{ S}}_{21}^{2} } \right] \, / \, \left[ {\left( {1 \, - {\text{ S}}11} \right)^{2} - {\text{ S}}_{21}^{2} } \right]} \right\} $$

2 $$ {\text{n}} = \left( {1/{\text{k}}_{0} {\text{d}}} \right) \, \left\{ {\left[ {\left[ {\ln \left( {e^{{{\text{ink}}}} 0^{{\text{d}}} } \right)} \right]^{\prime \prime } + 2{\text{m}}\pi } \right] - {\text{i}}\left[ {\ln \left( {e^{{{\text{ink}}}} 0^{{\text{d}}} } \right)} \right]^{\prime } } \right\} $$

3 $$ e^{{{\text{ink}}0{\text{d}}}} = {\text{ S}}_{21} / \, \left( {1 - {\text{S}}_{11} \left( {{\text{z}} - 1/{\text{z}} + 1} \right)} \right) $$

4 $$ \varepsilon = {\text{n}}/{\text{z}} $$

$$ \mu = {\text{nz}} $$

In Eq. (2), the symbols ′ and ″ denote the real and imaginary part operators, accordingly.

## Scattering and effective medium parameters of unit cell designs

**Figure 3:**
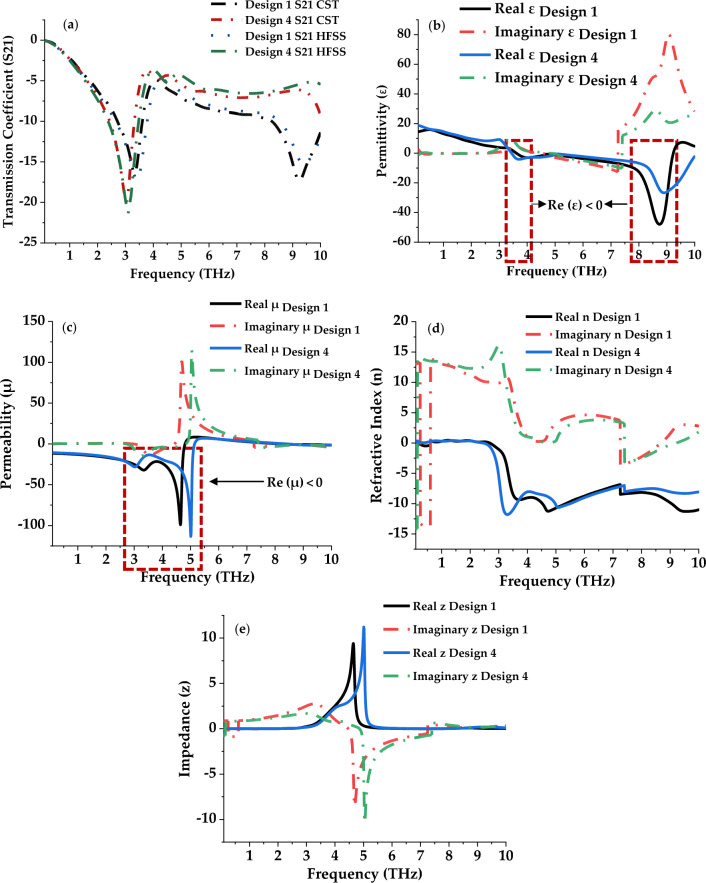
Scattering and effective medium parameters of the proposed designs: (**a**) S21; (**b**) Permittivity; (**c**) Permeability; (**d**) Refractive Index; (**e**) Impedance.

Figure 3 illustrates the scattering and effective medium properties of both symmetric square-shaped metamaterial designs. The S21 results of these metamaterial designs were plotted in Fig. 3a which was verified by adopting HFSS software. Both software gain almost equivalent resonance frequencies and magnitude values. For example, Design 1 exhibits responses at 3.32 and 9.24 THz through CST software, while, HFSS software produces 3.4 and 9.4 THz, respectively. On the other hand, Design 4 manifests single resonance at 3.03 THz and 3.10 THz from CST and HFSS software respectively. Therefore, only slight discrepancies occurred between these two analytical software which can be accepted for practical applications.

Moreover, the negative behaviour of permittivity (ℇ) values also occurred in both designs as demonstrated in Fig. 3b. For example, Design 1 manifests negative characteristics at the frequency ranges from 3.62–4.69 THz and 7.83–9.23 THz. Moreover, this design also exhibits negative permeability (µ) values at ranges from 2.98 to 3.59 THz, 4.01–4.71 THz, and 8.17–10.00 THz respectively. Meanwhile, Design 4 gained a single range ℇ and µ values at 3.45–5.59 THz and 0.1–5.13 THz, respectively (as shown in Fig. 3c. Theoretically, a material’s electrical conductivity and effective electron concentration are directly correlated with its negative permittivity behaviour^26^. It is commonly acknowledged that the free carrier density of materials can be successfully altered to modify the negative permittivity. In other words, negative permittivity behaviour in composite materials is highly related to carrier concentration, and low carrier concentration will result in weak negative permittivity. Metallic composites with high carrier concentrations are used in electromagnetic fields due to their high negative permittivity. The adopted silicon substrate material possesses a dielectric constant (ɛ) of 11.9 and a carrier concentration of 8.6 × 10^9^ cm^−3^. Furthermore, the refractive index (n) and the impedance values of the proposed designs were demonstrated in Fig. 3d,e. In this work, both proposed symmetric designs successfully manifest left-handed behaviours as shown in Table 2. Moreover, a single impedance peak point happened in each design likely at, 4.67 THz with 9.41 and 5.01 THz with 11.22, respectively.

**Table 2:** Left-handed behaviours for both Design 1 and 4.

Design	Frequency (THz)	ℇ	µ	n
1	3.62–4.80	− 0.11 to − 0.75	− 23.64 to − 0.39	− 9.24 to − 11.26
	8.17–9.23	− 17.67 to − 0.27	0 to − 0.83	− 8.21 to − 10.70
4	3.45–5.14	− 0.06 to − 0.54	− 13.99 to − 0.65	− 11.33 to − 10.57

## Parametric studies

In this section, several promising parametric analyses were carried out to gain the optimised performance in the proposed application frequency bands. For instance, the unit cell metamaterial was selected by adopting five different square-shaped metamaterial designs. These designs were constructed symmetrically by adopting square shape metamaterial copper design. Moreover, the chosen metamaterial designs undergo a few analyses such as clockwise rotation, array and layer structure designs, larger scale designs, novel design structure comparison and electric field distribution of the proposed design. In each investigation, the introduced designs exhibit unique and optimistic results.

### Selection process of unit cell design

**Figure 4:**
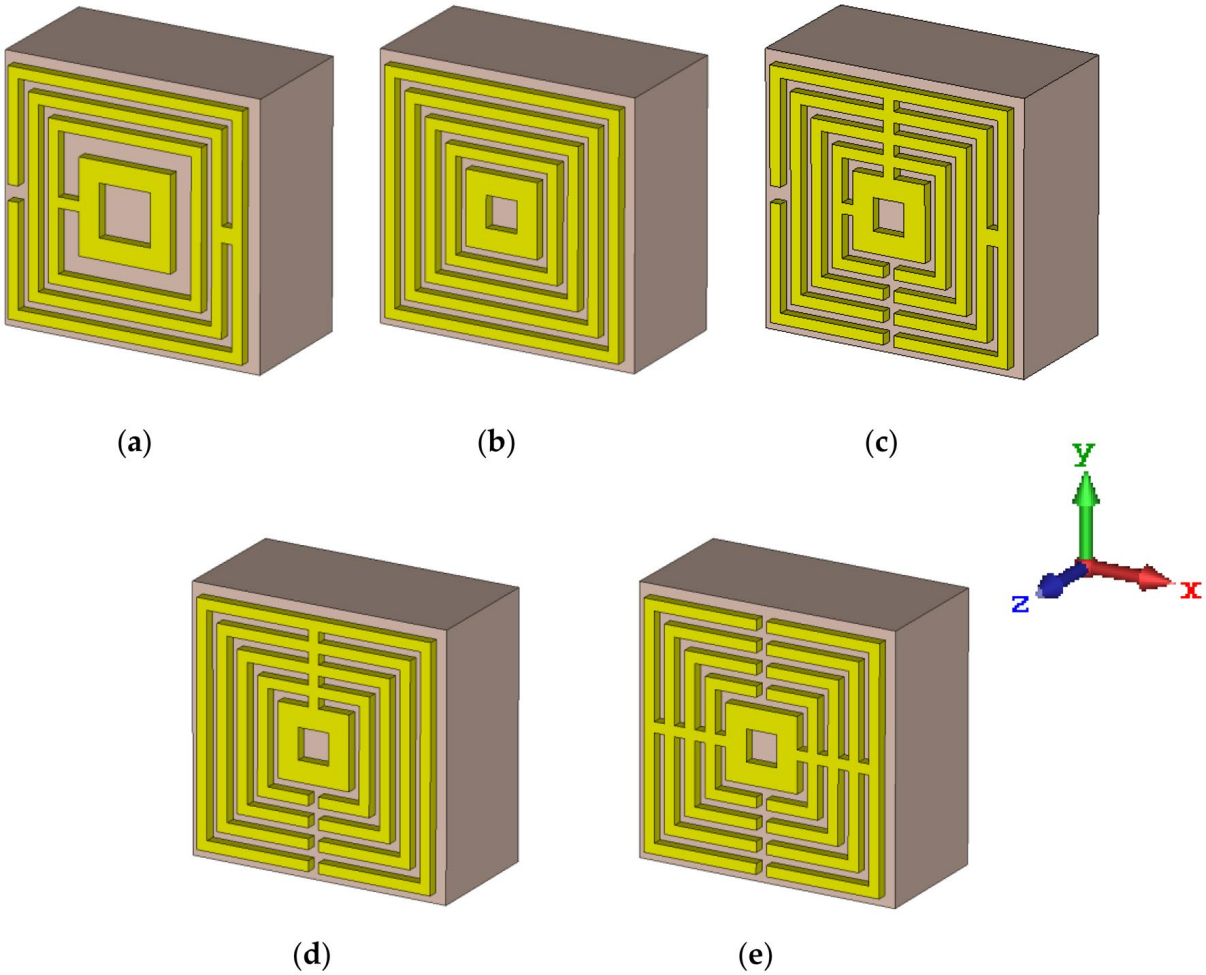
Introduced square-shaped metamaterial: (**a**) Design 1; (**b**) Design 2; (**c**) Design 3; (**d**) Design 4; (**e**) Design 5.

**Figure 5:**
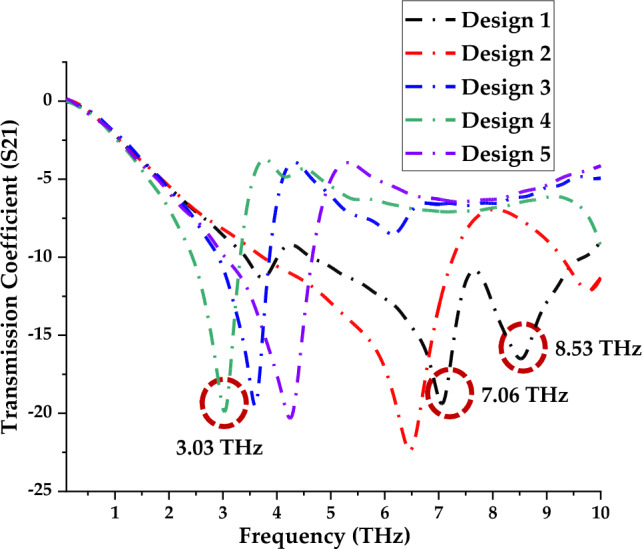
Transmission coefficient of the introduced five types of square metamaterial design.

According to our early investigation, the square-shaped metamaterial design exhibits lower band resonance frequencies than the other structures, particularly the circular shape designs. The fact that the square-shaped metamaterial typically induces an electromagnetic force around its structure, becomes an intriguing result that merits discussion. With the aid of external magnetic fields, this construction enables current to flow from one ring and into the next between the inner-ring spacing. Additionally, the square-shaped metamaterial essentially produces resonance frequencies at lower bands and has a higher current dispersion than conventional structures. It is noteworthy that this kind of composite structure features an additional capacitive coupling that aids in the manifestation of powerful distinctive behaviours. Therefore, five novel squared-shaped metamaterial design structures were proposed by adopting the simple trial and error method in this section. Hence similar types of substrate material and dimensions were adopted in this section to explore their scattering parameter properties. In conclusion, two types of square-shaped metamaterial designs with the optimised number of resonance frequencies between 0.1 and 10 THz were chosen in this selection process. In this investigation, several symmetric square-shaped metamaterial designs were proposed as illustrated in Fig. 4a–e. Moreover, the S21 results of the introduced designs were plotted as demonstrated in Fig. 5. Overall, the observation indicates that, single or double resonance response either at lower or higher terahertz frequency. For instance, Designs 3, 4 and 5 exhibit responses between the frequency of 2.5 THz to 4.5 THz. Moreover, the first two designs manifest S21 results between 6 and 9 THz. However, only Design 1 exhibited dual resonance frequencies such as 7.06 THz and 8.53 THz by adopting a compact substrate material. On the other hand, Design 4 successfully gains response at the lower frequency likely at 3.03 THz. The peak responses of both Designs 1 and 4 were highlighted by dotted circles in Fig. 5. From these results, it is clear that adding multiple connecting bars between rings and split gaps can gain lower resonance frequency besides only increasing the number of rings. However, the construction of more connecting bars and split gaps in a design will cause the resonance frequency to shift to a higher range as shown in Fig. 5. Therefore, the main constraint that the researcher will face when handling compact-sized metamaterial design is generally due to the desired resonance frequency and their performances.

Typically, not all square-shaped split ring resonator metamaterial structures will always manifest optimised outcomes. Hence, modification of several parameters likely, split gaps, connecting bars and the number of rings on the design, especially in compact structure can gain desired performances. The trial and error method is generally used to effectively save time due to its simple and easy technique instead of utilising new substrate material in the market or synthesising new material which is relatively costly. Therefore, the work mainly focused on obtaining superior performance by adopting a compact and novel design composed of existing and easily available substrate material. From this analysis, two types of metamaterial design structures, such as Design 1 and Design 4 were adopted to further investigate the performances through several parametric studies. For instance, clockwise rotation, array and layer structure designs, larger scale, novel design structure comparisons and electric field distribution were analysed in this work. All these studies were analytically simulated by focusing on the performances of the transmission coefficient.

### Clockwise rotation

**Figure 6:**
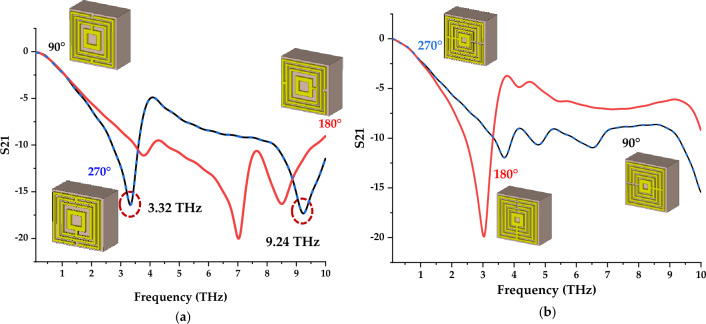
Transmission coefficient of results at various clockwise rotations such as 90°, 180° and 270° for (**a**) Design 1; (**b**) Design 4.

In this parametric study, the adopted two types of symmetric metamaterial designs were rotated clockwise about 90° each cycle. This observation clearly shows dramatic changes that occurred in S21 results when Design 1 was rotated 90°. For instance, the design initially exhibits dual resonance frequencies after 7 THz, while the rotated structure manifests dual responses at 3.32 THz and 9.24 THz, respectively (as highlighted in dotted red circles in Fig. 6a). Moreover, the 90° and 270° rotated structures exhibit similar results for both proposed designs. Meanwhile, both 180° and the initial design possess similar responses. Overall, Design 4 did not exhibit unique behaviour when the design rotated clockwise as shown in Fig. 6b. Hence, the rotated 90° design was assigned as Design 1 hereafter for further examination. Overall, this method obtained reasonable changes only for Design 1 when compared to the initial designs.

### Array and layer structure design

**Figure 7:**
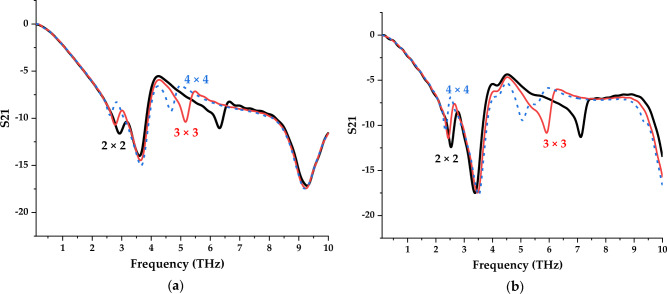
Transmission coefficient of results of various array structures for (**a**) Design 1; (**b**) Design 4.

**Figure 8:**
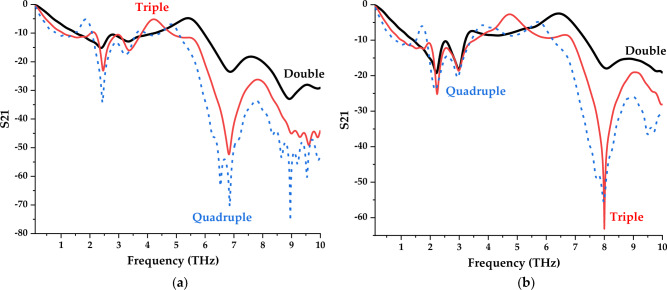
Transmission coefficient of results of various layer structures for (**a**) Design 1; (**b**) Design 4.

Figures 7 and 8 illustrate the S21 results of the array and multilayered metamaterial by adopting both symmetric designs. The proposed designs were arranged in 2 × 2, 3 × 3, and 4 × 4 cells, while double to quadruple layers were adopted for multilayer analysis. Due to the size constraint variable, the array cell was used until the overall size reached 20 µm. The array cell results tie well with previous studies wherein only slight differences occurred in both designs where the new responses mostly possess less than a − 10 magnitude value. The 2 × 2 array cell metamaterial structure manifests almost similar resonance response compared to unit cell design. Although the number of array cells increases causing the occurrence of more than two resonance frequencies, the peak point is limited in terms of the magnitude values. For example, Design 1 in the 3 × 3 array cell as demonstrated in Fig. 7a manifests a new peak point near 5 THz, but it only has a magnitude value less than − 12.5. This same phenomenon occurred for Design 4 as shown in Fig. 7b.

On the other hand, the multilayer metamaterials indicate promising results when compared to the array cell analysis. Design 1 in quadruple layers manifests superior resonance frequencies when compared to the rest of the layers as illustrated in Fig. 8a. For instance, five resonance frequencies exhibited in this design at 2.44, 6.54, 6.85, 8.96, and 9.53 THz. Moreover, the double and triple-layer metamaterial designs manifest a similar number of peak points between the ranges of 0–3 THz and 6–10 THz, respectively. Furthermore, Design 4 in the quadruple layer exhibits resonance frequencies with more than − 15 magnitude values at 2.17, 2.95, 7.99 and 9.49 THz as shown in Fig. 8b. Meanwhile, three acceptable peak points happened for triple-layer metamaterial design, likely at 2.24, 2.99 and 8.00 THz. Moreover, the double-layer metamaterial has responses with magnitude values less than zero, for example at 2.21, 2.98 and 8.08 THz. However, even better results are achieved when the dimension of the substrate material is increased as described in the following subsection.

### Larger scale

**Figure 9:**
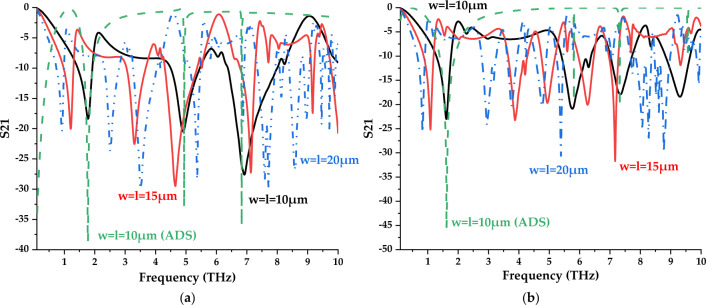
Transmission coefficient of various larger scale metamaterial structures for (**a**) Design 1; (**b**) Design 4.

**Figure 10:**
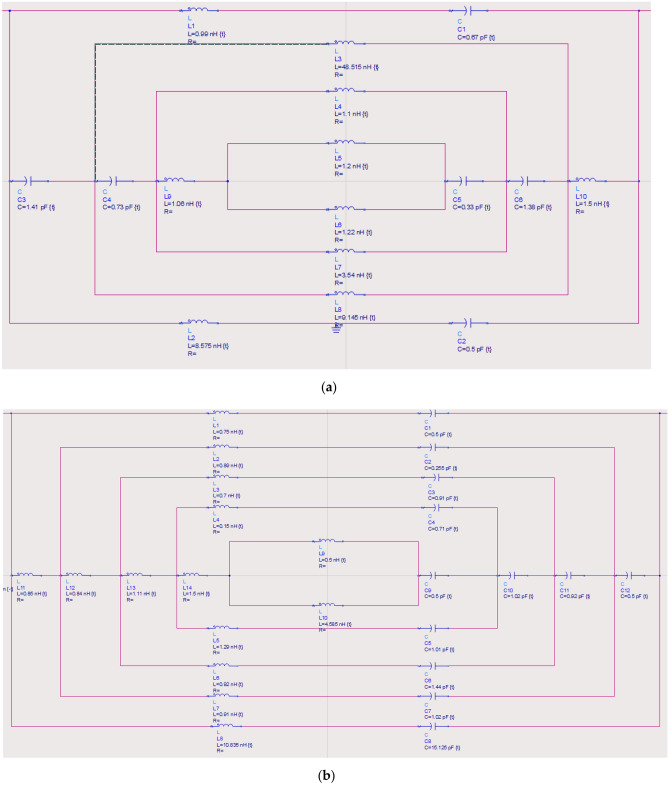
Equivalent circuit model adopted from ADS software of (**a**) Design 1; (**b**) Design 4.

The selected two types of unit cell designs went through scaling operations in CST software to enlarge the dimension of the initially proposed design. Three scales were adopted in this parametric study likely 2, 3 and 4 where the maximum dimension of substrate material reaches 20 µm. As mentioned earlier, the bigger unit cell design typically exhibits better and multiple resonance.

frequencies. The S21 results as illustrated in Fig. 9a, b prove this theory and the biggest unit cell design reaches ten resonance frequencies between the range of 0.1 to 10 THz. Unlikely the array cell analysis in the previous subsection, these larger-scale examination exhibits extraordinary S21 results in both selected Design 1 and 4. Most of the resonance peaks in each scale possess magnitude values of more than -15. In a nutshell, this analysis indicates that the S21 of the compact symmetric designs can be altered by increasing the overall size likely from 5 µm to 10, 15 and 20 µm, respectively. Although these designs are very suitable for many applications, this work strictly focuses on compact-sized metamaterial analysis since the miniaturisation concept has been widely adopted past few decades. This is because the new technological devices or equipment are constructed in smaller sizes based on the market demand. Hence, this work aims to introduce optimised performances by simply utilising easily available materials instead of synthesising new material which will cost highly and take time. Overall, the arrangement of the square split resonators also influences the responses such as Design 1 manifest 3, 6, and 11 peak points, while Design 4 exhibit 4, 5 and 10 responses when the dimension increases. On the other hand, both scale 2 metamaterial designs were selected to further validate the S21 results by adopting ADS software. The equivalent circuit models of the two distinct metamaterials were constructed as demonstrated in Fig. 10a,b. The observation indicates that Design 1 possesses a total of 6 capacitive effects and 10 inductive effects. Moreover, Design 4 exhibits 12 capacitive and 14 inductive effects with distinct values. The evaluated S21 results from this software were compared with the initial data from CST software as illustrated in Fig. 9a,b. The comparison revealed slight discrepancies between resonance frequencies that occurred for Design 1, while Design 4 exhibited a major difference in the last peak response compared to the rest of the resonance frequencies. However, both designs manifest narrow S21 responses through ADS software when compared to results from CST software.

### Novel design structure comparison

**Figure 11:**
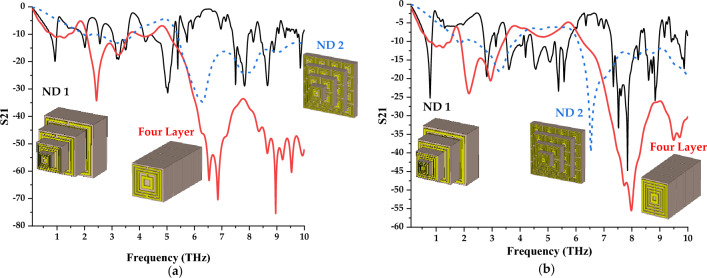
Transmission coefficient of novel design structure (**a**) Design 1; (**b**) Design 4.

A comparison of several novel arrangements of the proposed symmetric designs is explored in this subsection as shown in Fig. 11a,b. First of all, the unit cell designs are arranged based on the dimension assigned as ND 1. For this structure, four different dimensions were adopted such as 5, 10, 15 and 20 µm. All these unit cells are aligned at the centre and arranged one by one as a layer form. The same concept was adopted for ND 2 structure as well, but the combination of unit and array cells was used to construct this novel design. Together with these structures, the four-layer design is also compared to investigate the performance changes. Although the larger unit cell exhibits better resonance frequencies as discussed previously, in this analysis, the proposed designs also exhibit lower and higher responses in both selected designs. For example, ND 1 structure by adopting Design 1 as shown in Fig. 11a manifests 8 very narrow peak points. Meanwhile, ND 1 by adopting Design 4 exhibits 11 resonance frequencies as demonstrated in Fig. 11b. Besides that, the multilayer structures possess better magnitude value responses when compared to the novel design structures. In conclusion, the S21 properties of a distinct metamaterial design can be successfully altered by changing the physical properties instead of the chemical composition.

### Electric-field distribution

**Figure 12:**
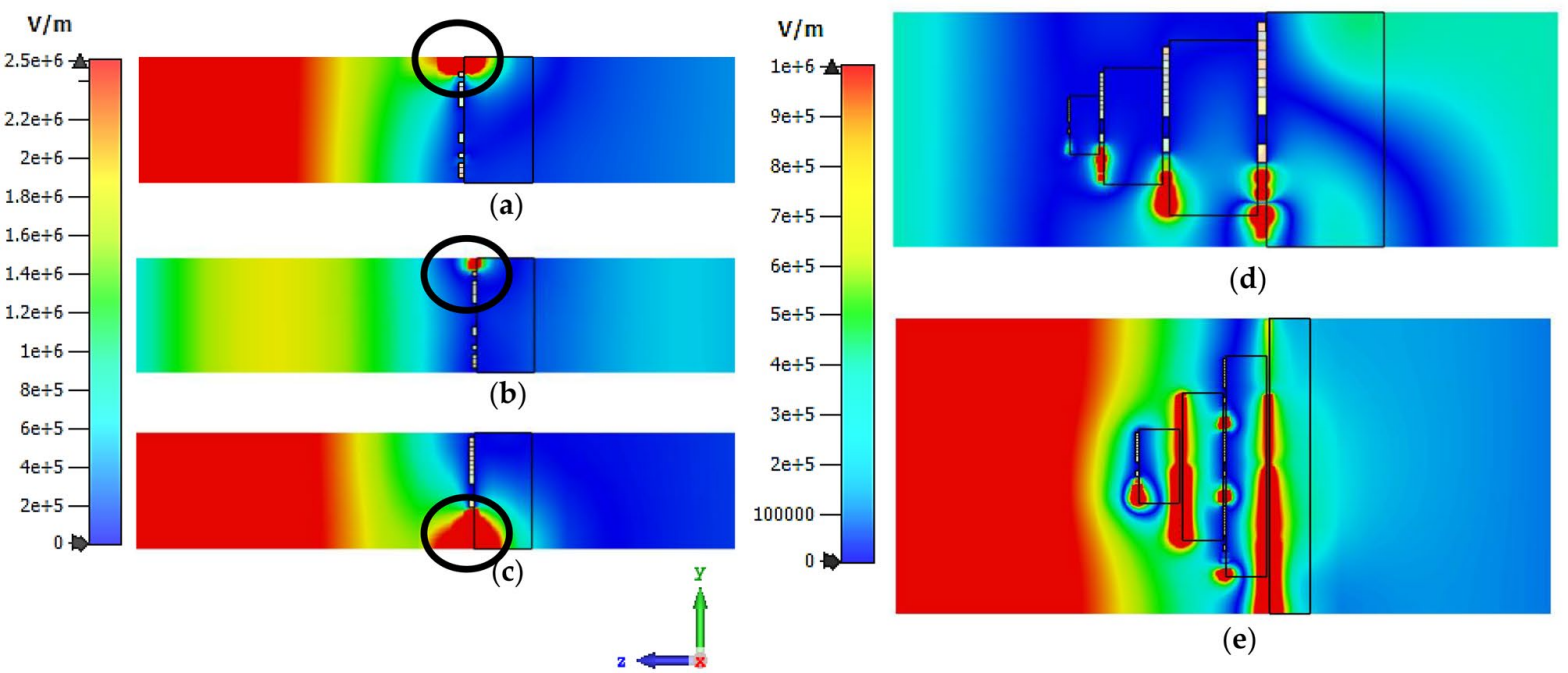
Electric field distribution of symmetric design (**a**) Design 1 at 3.32 THz; (**b**) Design 1 at 9.24 THz, (**c**) Design 4 at 3.03 THz, (**d**) ND 1 Design 4 at 3.03 THz, (**e**) ND 2 Design 4 at 3.03 THz.

The electric field distribution of the symmetric square-shaped metamaterial designs at each resonance frequency was explored in this section as illustrated in Fig. 12a–e. The figures demonstrate the wave propagation through the designs in contour absolute by adopting the yz cutting plane. The observation indicates that unit cell designs produce intense field distribution on the split gaps of the metamaterial structure. For instance, in Design 1, the electric field focuses on the upper hand, while in Design 4 localised on the lower hand as highlighted in black circles in Fig. 12a–c. On the other hand, ND 1 and ND 2 by adopting Design 4 were selected to compare the electric field distribution with the unit cell designs as shown in Fig. 12d,e. Overall, the bigger structure, for instance, ND 2 which is composed of array cells with a maximum dimension of 20 µm × 20 µm able to produce high electric field distribution localised on every layer when compared to ND 1.

### Absorption properties

**Figure 13:**
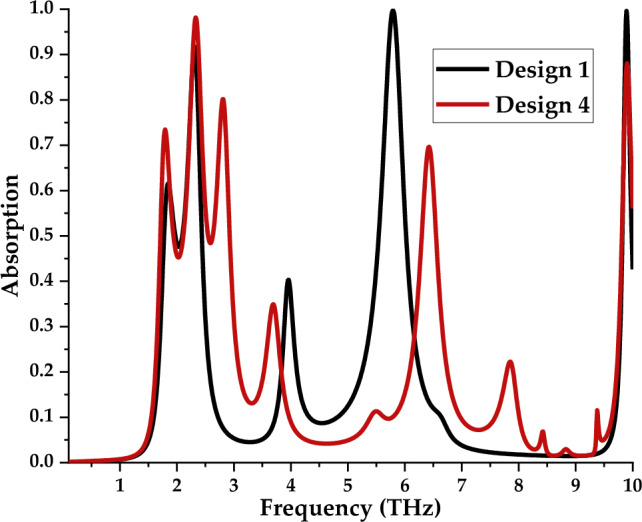
Absorption performance of the metamaterial designs with scale 2.

Both Design 1 and 4 with scale 2 were adopted to explore and analyse the absorption properties as shown in Fig. 13. The observation indicates unique absorption behaviour occurrence for both designs with distinct resonance frequencies. For instance, Design 1 exhibits four peak values that have more than 0.5 absorptions at 1.84 THz, 2.30 THz, 5.79 THz, and 9.89 THz, respectively. Meanwhile, Design 4 manifests 5 peak responses at 1.79 THz, 2.33 THz, 2.81 THz, 6.43 THz, and 9.90 THz, respectively.

## Conclusions

The paper concludes by arguing that, the proposed compact symmetric design effectively examined their performances on the scattering and effective medium parameters at the frequency range from 0.1 to 10 THz. Two distinct square-shaped metamaterial designs were introduced which possess double and single resonance responses. Overall, several parametric studies were successfully explored and analysed in this research work. The changes in physical properties by adopting existing materials led to extraordinary performances. Therefore, this will typically save time instead of synthesising new material to apply it for the proposed application. This proves that not only new materials but the existing materials with a distinct combination of metamaterial designs by adopting a simple trial and error method can encourage unique outcomes. In conclusion, symmetric split ring resonator metamaterial has the potential to be used in a variety of applications, including lenses, biomedical detection, and metamaterial absorbers, but focusing on sensing applications.

## Data Availability

All the data are available within the manuscript.
